# A Flexible System for Cultivation of *Methanococcus* and Other Formate-Utilizing Methanogens

**DOI:** 10.1155/2017/7046026

**Published:** 2017-11-19

**Authors:** Feng Long, Liangliang Wang, Boguslaw Lupa, William B. Whitman

**Affiliations:** Department of Microbiology, University of Georgia, Athens, GA 30602-2605, USA

## Abstract

Many hydrogenotrophic methanogens use either H_2_ or formate as the major electron donor to reduce CO_2_ for methane production. The conventional cultivation of these organisms uses H_2_ and CO_2_ as the substrate with frequent replenishment of gas during growth. H_2_ is explosive and requires an expensive gassing system to handle safely. Formate is as an ideal alternative substrate from the standpoints of both economy and safety but leads to large changes in the culture pH during growth. Here, we report that glycylglycine is an inexpensive and nontoxic buffer suitable for growth of *Methanococcus maripaludis* and *Methanothermococcus okinawensis*. This cultivation system is suitable for growth on liquid as well as solid medium in serum bottles. Moreover, it allows cultivation of liter scale cultures without expensive fermentation equipment. This formate cultivation system provides an inexpensive and flexible alternative for the growth of formate-utilizing, hydrogenotrophic methanogens.

## 1. Introduction

Methanogens are strictly anaerobic microorganisms belonging to the *Euryarchaeota*. As a large and diverse group, they are distinguished by their capability to obtain most if not all of their energy for growth from methane production or methanogenesis [1]. In general, methanogens only utilize a limited number of substrates for methanogenesis, such as CO_2_; H_2_; formate; methyl-group containing compounds such as methylamines, methylsulfides, and methanol; acetate; and a few low molecular weight alcohols. They do not use sugars, amino acids, or most other common organic substrates [2]. Most methanogens are hydrogenotrophs that use H_2_ as the primary electron donor to reduce CO_2_ to methane. Many hydrogenotrophic methanogens can also use formate as the major electron donor [2]. As shown in (1), four molecules of sodium formate are oxidized, yielding one molecule of methane and three molecules of CO_2_.

(1) $$\begin{array}{c} 4HCOONa+2H_{2}O\to {CH}_{4}+3CO_{2}+4NaOH. \end{array}$$

Because no more than one ATP is formed per mol of CH_4_ [3], relatively large amounts of formate are required for even modest growth. Growing cells with sodium formate also leads to a significant accumulation of NaOH, which raises the pH of the medium and inhibits growth. For methanococci, the alkaline pH also causes cell lysis and rapid killing [4]. As a result, pH control becomes a critical concern when cultivating methanogens with sodium formate.

One solution is to titrate the rise in pH with formic acid during growth in a fermenter [5]. For growth in culture tubes and plates, the medium pH can also be controlled with a built-in formic acid reservoir [4]. This cultivation system uses a 6 × 55 mm acid reservoir containing 200 *μ*L formic acid to stabilize the medium pH [4]. As the pH increases, the absorption of formic acid from the headspace also increases, maintaining the pH within levels that support growth. Although this method allows good growth on formate-containing medium, its requirement for manual dexterity precludes it from routine use. Using formate as substrate has also been established in a chemostat system. Costa et al. [6] used formate to grow *M. maripaludis* in chemostat for studying the transcriptional regulation. The sodium formate was added at 0.38 M, while the pH was maintained at 6.95 by automatic addition of 10% (*v*/*v*) H_2_SO_4_ [6]. The cell density and growth rate achieved with either formate or H_2_/CO_2_ were the same during chemostat cultivation [6–9].

During growth of *M. maripaludis* with formate, formate dehydrogenase (Fdh) is the key enzyme for formate utilization. Fdh is encoded by two sets of genes, *fdhA1B1* and *fdhA2B2* in *M. maripaludis* [10]. Lupa et al. [11] found that mutants with deletions in *fdhA1* grew poorly on formate only after an extended lag. In contrast, mutants with deletions in *fdhA2* grew nearly the same as wild-type. Because of this and other evidence, Fdh1 was proposed to play a major role in fomate utilization [11].

Over the past decade, many genetic methodologies have been developed in *M. maripaludis*. These include effective selectable genetic markers [12–16], multiple plasmid shuttle vectors [17], high-efficiency transformation [18], direct gene replacement mutagenesis [19], markerless gene deletion systems [20], random mutagenesis [21], in vivo transposon mutagenesis [22, 23], reporter genes technologies [24, 25], and chemostat cultivation [6–9]. Thus, genetic manipulation of *M. maripaludis* is easy and effective, and these approaches have become powerful tools to study the metabolism and physiology of multiple *Methanococcus* species. However, the requirement for H_2_ growth limits the ability of these genetic tools to be widely applied in laboratories that do not have established systems for handling H_2_ gas.

Here, we report a medium to cultivate the mesophilic, marine species *M. maripaludis* on formate using glycylglycine buffer as the pH stabilizer. Ordinarily, *M. maripaludis* is cultivated in aluminum-sealed tubes with 5 mL of medium under H_2_/CO_2_ mixture (4 : 1, *v*/*v*) at 276 kPa [26]. For comparison, in our formate cultivation system, the pressure is reduced to 104 kPa, allowing use of more inexpensive stoppers. In addition, frequent gas refilling is avoided without greatly sacrificing growth yield. Simple modifications of common glassware also allows liter-scale cultivation using only a gassing station and a vacuum pump. In addition, the solid medium has a high plating efficiency suitable for genetic experiments. With minor adjustment in medium composition, the procedure is also suitable for growth of the extreme thermophile *Methanothermococcus okinawensis.*

## 2. Materials and Methods

### 2.1. Strains, Media, and Growth Conditions

*Methanococcus maripaludis* strain S2 was obtained from our laboratory collection (Whitman et al.) [27] and cultured at 37°C. *Methanothermococcus okinawensis* strain IH1 was obtained from Takai et al. and cultured at 62°C [28].

Cultures were grown in H_2_/CO_2_ medium (McNA, a minimal medium with 10 mM sodium acetate) or formate medium (McF) reduced with 3 mM cysteine hydrochloride. The 5 mL cultures were grown in 28 mL aluminum-sealed tubes. For McNA, the tubes were pressurized to 276 kPa with H_2_/CO_2_ (4 : 1, *v*/*v*) and refilled with the same gas every 24 hours after inoculation. Detailed protocols for growth on formate are given in Appendix A. Briefly, McF medium contained 0.4 M sodium formate and was buffered with 0.2 M glycylglycine (pH = 8.0). The medium was first sparged with N_2_ to remove most of the O_2_, and 3 mM cysteine chloride was then added. Tubes were pressurized to 103 kPa with N_2_/CO_2_ (4 : 1, *v*/*v*) before autoclaving. Prior to inoculation, 3 mM sodium sulfide was added as the sulfur source.

The buffers tested were obtained from Sigma Chemical Co. and included (with the counter ion) Tricine/NaOH (N-[Tris(hydroxymethyl)methyl]glycine), Bicine/NaOH (*N*,*N*-bis(2-hydroxyethyl)glycine), Tris/HCl (2-amino-2-hydroxymethyl-propane-1,3-diol), glycine/NaOH, and glycylglycine/NaOH. During formate medium preparation, ingredients were added as listed in the appendices, and the organic buffers were added from stock solutions at pH 7. The concentration of NaCl was adjusted depending upon the amount of sodium formate and sodium in the buffer used so that the final concentration of sodium ion was 0.4 M.

The final medium was also tested for plating (Appendix B) and growth of 1.5 L cultures (Appendix C).

### 2.2. Rapid Protocol for Preparation of Formate Medium

After combining the components of McF medium, cysteine was added and the medium was dispensed into culture tubes on the bench without anaerobic precautions (Appendix D). Without delay, the tubes were sealed with stoppers and aluminum seals. The tubes were then connected to a gassing manifold, and the air was removed by three successive cycles comprising 45 seconds of vacuum followed by 15 seconds of 104 kPa N_2_: CO_2_ (4 : 1, *v*/*v*). After exchanging the gas, the medium was autoclaved for 20 min with rapid exhaust. For the control medium, the medium was dispensed in an anaerobic chamber as described in Appendix A, and the gas was exchanged for three cycles with N_2_/CO_2_ (4 : 1, *v*/*v*) prior to autoclaving.

## 3. Results

### 3.1. Optimization of the Formate Medium and Growth Conditions

**Figure 1:**
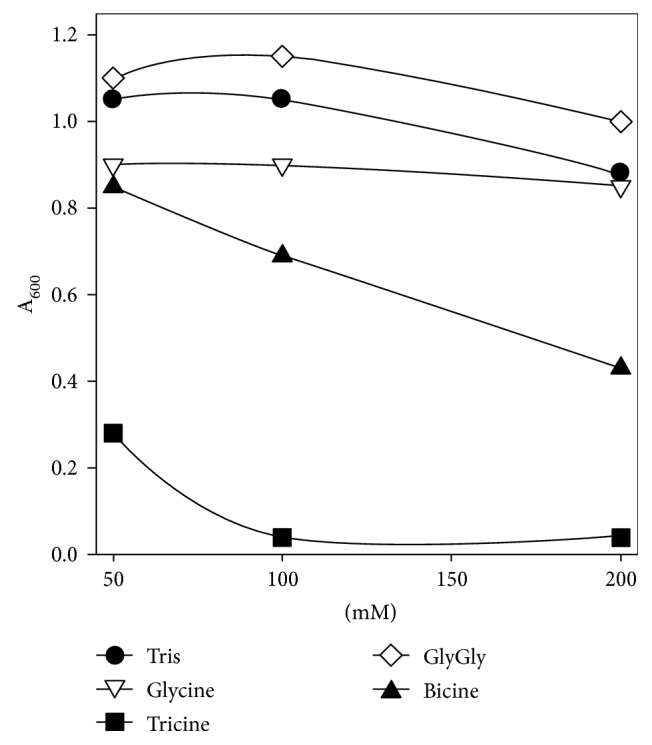
Effect of selected buffers on growth. *M. maripaludis* S2 was grown in McN (H_2_/CO_2_) medium with different concentrations of tested buffers. The culture absorbance was determined after one day.

To determine if organic buffers were inhibitory for growth, they were added to the medium during growth of *M. maripaludis* on H_2_/CO_2_. Because the medium was strongly buffered with bicarbonate and CO_2_, the buffers did not affect the initial pH. Under these conditions, Tricine was strongly inhibitory (Figure 1). While glycine and Bicine had little effect on cell yield, both increased the lag phase at higher concentrations (data not shown). In contrast, Tris and glycylglycine were not inhibitory and resulted in moderate decreases in the lag phase, presumably by maintaining an optimal pH during the early growth phase (data not shown). Therefore, Tricine and Bicine were omitted from further experiments.

**Figure 2:**
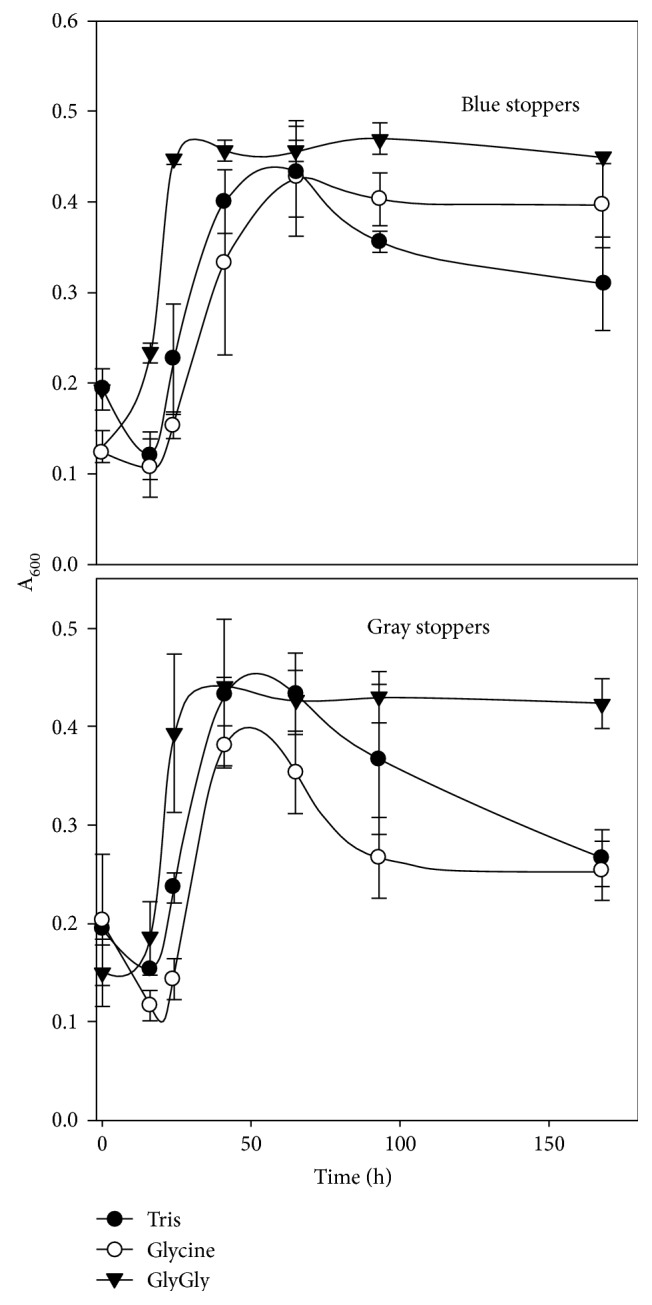
Growth of *M. maripaludis* S2 with 200 mM formate and 100 mM of Tris, glycine, and glycylglycine buffers. Two kinds of serum bottle stoppers were used. Blue stoppers are thick butyl rubber stoppers (Bellco Glass Inc., Vineland, NJ, cat. number: 2048-11800, 704.82 USD/1000). They are commonly used for H_2_/CO_2_ medium. Butyl rubber gray stoppers (Wheaton Science Products, cat. number: W224100-202, 174.2 USD/1000) were also tested for their durability during *M. maripaludis* cultivation.

Tris, glycine, and glycylglycine were further tested for their buffering capacity during growth with 200 mM sodium formate. In the presence of 100 mM buffer, the culture reached a maximal absorbance of about 0.4–0.45 after 20 h (Figure 2). During the first two days of incubation at 37°C, all three buffers maintained the medium pH around 7.2–7.6. However, during extended incubations, decreased absorbance and cellular lysis were observed in media buffered with Tris and glycine (Figure 2, data not shown). In contrast, the absorbance of cultures supplemented with glycylglycine remained stable for six days at 37°C (Figure 2). Moreover, in glycylgylcine-buffered medium, the culture absorbance did not change for up to six weeks at room temperature, and it was still possible to transfer stock cultures to fresh medium. Cultures in McF medium were also used to prepare −80°C freezer stocks in 30% (*v*/*v*) glycerol [26, 29], and these cultures retained viability for at least five years.

To reduce the cost of anaerobic medium preparation, the influence of different types of stoppers on growth was also tested. Cultivation on H_2_/CO_2_ is usually performed at 276 kPa in 28 mL aluminum-sealed tubes. For this reason, thick butyl rubber stoppers (Bellco Glass Inc., Vineland, NJ, cat. number: 2048-11800) are commonly used. These stoppers are made to minimize gas leakage and sustain multiple needle stabs during medium preparation, inoculation, and sampling. As an alternative, butyl rubber grey stoppers (Wheaton Science Products, cat. number: W224100-202) are much less expensive although thinner. Although these stoppers cannot maintain high pressure, they might be suitable for growth on formate at lower pressure. As shown in Figure 2, Wheaton stopper-sealed cultures showed comparable growth profiles and stability, especially in medium supplemented with glycylglycine. In contrast, white precipitates were observed in cultures supplemented with Tris and glycine (data not shown). The composition of the medium resembles that of seawater and contains high levels of divalent cations. During autoclaving, the pH of this medium increases due to the reduced solubility of CO_2_ at high temperatures. Presumably, these precipitates represent phosphate salts that become insoluble at alkaline pH. The precipitates were rarely observed following autoclaving with the thicker stoppers, probably because they retained CO_2_ better during autoclaving.

**Figure 3:**
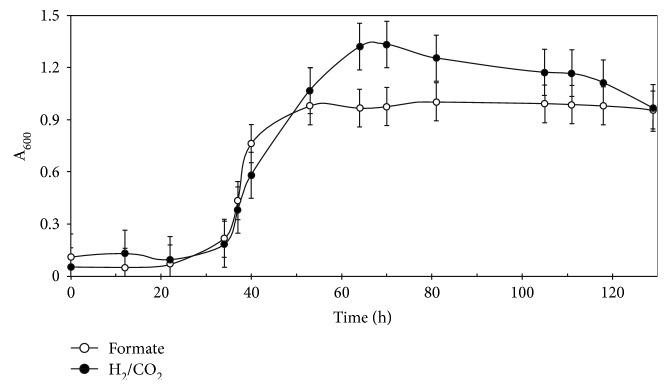
Growth of *M. maripaludis* S2 in H_2_/CO_2_ (●) and formate medium (○). The inoculum size was 5 × 10^4^ cells per 5 mL of culture. All values were the averages of five cultures.

In the presence of 100 mM glycylglycine, the growth yields increased with formate concentrations in nonlinear fashion and were maximal at 0.6 M. Growth was inhibited with 1 M sodium formate, presumably due to sodium toxicity (data not shown). At high formate concentrations and 100 mM glycylglycine, cells lysed in the stationary phase, presumably due to alkalinization of the medium. Increasing the glyclyglycine concentration to 200 mM with 0.4 M formate was found to be optimal for batch growth. In this condition, the growth rate was similar to that in H_2_/CO_2_ medium. Moreover, the maximum OD_600 nm_ of 1.0 was comparable to 1.4 in H_2_/CO_2_ medium (Figure 3). Thus, the cellular yields per mole of electron donor were nearly equivalent. For instance, medium with 0.4 M formate contained about 2 mmol of formate in 5 mL, and the growth yield was about 340 mg dry wt L^−1^ or 0.85 g dry wt mol^−1^ of formate. For 5 mL H_2_/CO_2_ cultures with 2.7 mmol of H_2_, the growth yield was about 400 mg dry wt L^−1^ or 0.74 g dry wt mol^−1^ of H_2_.

Good growth was also found on formate medium containing 1.0% (*w*/*v*) agar in serum bottles. Details on preparation are given in Appendix B, but it is similar to the protocols described earlier [30, 31]. Similar to growth with H_2_/CO_2_ medium, isolated colonies appeared after 3 to 5 days of incubation, and the plating efficiency was 100%.

### 3.2. A Simple Medium-Scale Cultivation System for *M. maripaludis* and *M. okinawensis* with Formate

**Figure 4:**
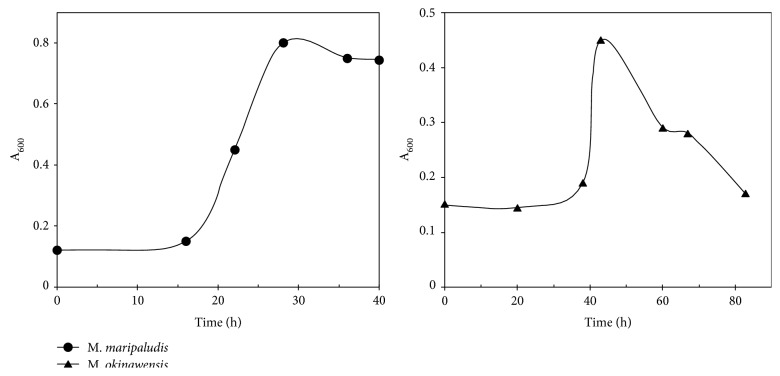
Growth of *M. maripaludis* S2 (●) and *M. okinawensis* (▲) in the medium-scale culture system. The inoculum was 10^10^ cells per 1.5 L of culture. All values are the averages of three cultures.

The modified formate medium was also useful for cultivation of *M. maripaludis* and *M. okinawensis* at liter or medium-scale for the preparation of biomass for enzyme and other studies. For this purpose, a simple cultivation system was developed using common lab glassware and equipment (Appendix C). Comprised largely of a 2 L cultivation bottle, a water trap, and a gas trap, each assembly supported growth of 1.5 L of culture. During growth, the exhaust line allowed the CH_4_ and CO_2_ formed to escape, the water trap prevented backflow of water into the culture, and the gas trap prevented back diffusion of air into the culture bottle. A protocol was also developed to ensure complete reduction of the medium before inoculation (Appendix C). Although the medium was sparged prior to inoculation, no gassing was required after inoculation, and the system could be easily moved to fume hood, incubator, or some other well-ventilated space. With a 2% inoculum, *M. maripaludis* S2 grew to about OD_600 nm_ = 0.8 after 15 hours of incubation at 37°C. In the same medium, *M. okinawensis* IH1 grew to an OD_600 nm_ = 0.6 (Figure 4). However, reduction of the pH of the glycylglycine buffer stock solution to 6.5 reduced the lag phase of *M. okinawensis* to 12 h at 62°C without reducing the yield (data not shown). For both cultures, the cell yield was around 1 g (wet weight) per L.

### 3.3. Rapid Preparation of Medium without an Anaerobic Chamber

An anaerobic chamber is often used for preparing medium for methanogens, but it is expensive and occupies a large amount of laboratory space. To determine if the formate medium could be prepared in laboratories with limited anaerobic equipment, it was prepared aerobically, and the gas was exchanged with a vacuum pump and gas line connected to a simple gassing manifold controlled by a three-way ball valve. The system was constructed from standard compression fittings so that its fabrication required little equipment and no special expertise. It was designed so that ten tubes or serum bottles could be prepared at one time. A vacuum pressure gauge was used to monitor the gas. After dispensing the medium aerobically, gassing/vacuum cycles were performed to remove O_2_ from the medium (Appendix D). Interestingly, growth in medium with even one gassing/vacuum cycle was nearly the same as in conventionally prepared medium (data not shown). Cultures of *M. maripaludis* are often tolerant to O_2_, and growth of log phase cultures is unaffected by O_2_ partial pressures < 20 kPa according to our experience. Therefore, it was possible that the large size of inoculum may have protected cells from residual O_2_. To examine the suitability of this method for small inocula, a most probable number (MPN) experiment was performed in medium prepared with three cycles of gas exchange (Table 1). The most probable numbers were 50 and 160 in media prepared by the rapid or standard protocol with an anaerobic chamber, respectively [32]. These high numbers were not significantly different and would only be possible if growth could be initiated by only one or two cells in both media.

This protocol was also suitable for preparation of solid medium and plating for isolation of mutants or other clonal cultures (Appendix D). Agar slabs were formed in serum bottles as described in Appendix B. After growth, single colonies were picked with a syringe needle and transferred to broth under a stream of N_2_ gas.

## 4. Discussion

The medium and culturing system for methanogens developed here attempted to address multiple concerns. First, the reagents and equipment should be accessible to many research laboratories. The replacement of H_2_ with formate as the major substrate for methanogenesis removed the need for a H_2_ handling system, reducing the cost as well as increasing the safety of culturing. The cost of medium preparation can be further reduced by using much less expensive septum stoppers. Moreover, a simple gassing manifold was sufficient, and an anaerobic chamber was not needed. These methods are straightforward and do not require extensive training. At the University of Georgia, this culturing system was widely used by undergraduate students to isolate and cultivate mutants of *M. maripaludis*. While training is still required, especially for the safe use of syringes and pressurized glassware, many of the elaborate manipulations of the Hungate method [33] are avoided. For many biological investigations, it is often necessary to generate cultures from single cells as well as generate large amounts of biomass. Both of these are often difficult with fastidious anaerobes. The system developed here had a high plating efficiency, and it was possible to develop cultures from only a few cells. Therefore, it is suitable for the isolation of mutants or other genetic experiments. In addition, it was possible to generate sufficient biomass for enzymatic assays and other biochemical analyses. The glycylglycine buffer prevented alkalinization of the medium and allowed the cultures to remain viable for several weeks on the bench. The addition of glycerol allowed maintenance of viable cultures for at least five years at −80°C. Nevertheless, the formate medium allows a similar growth rate and cellular yield as H_2_/CO_2_ medium. Moreover, this medium and protocol were adapted by Weimar et al. [34] for a multiwell plate method to screen chemical compound libraries [34]. *M. maripaludis* was grown in 96-well microtiter plates sealed in an AGS AnaeroGen compact bag (Oxoid) and incubated at 37°C inside an anaerobic chamber containing 5% H_2_, 5% CO_2_, and 90% N_2_ [34]. Therefore, these methods can be readily adapted for a number of experimental approaches.

## Figures and Tables

**Figure 5:**
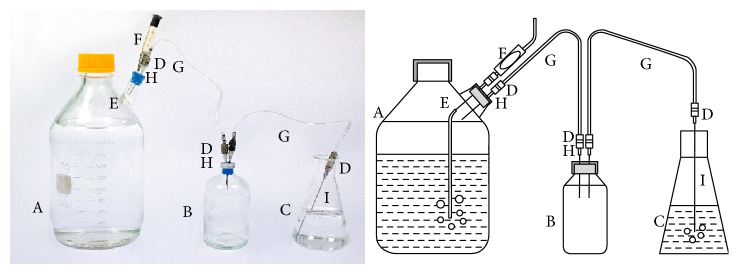
Medium-scale cultivation system with culture bottle, sparging, and exhaust. The cultivation system comprises a 2 L cultivation bottle (A), water trap (B: 160 mL serum bottle with a blue butyl stopper and an aluminum seal), and a gas trap (C: 150 mL Erlenmeyer flask). The cultivation bottle is constructed from a Pyrex bottle (cat. number: 06-414-1E) with the top of a 28 mm aluminum seal tube attached as a side arm. The side arm is sealed with a blue butyl rubber stopper without the aluminum seal (Bellco Glass Inc., Vineland, NJ, cat. number: 2048-11800). Leaving off the aluminum seal allows the stopper to pop off and release any pressure that might form if the exhaust line becomes clogged. The gassing line (D, E, and F) is for sparging the medium after autoclaving and is assembled with F, a 2-cc interchangeable glass-filter syringe barrel (Micro-Mate™, cat. number: 14-825-1B) packed with cotton and sealed with a number 00 rubber stopper (Balch and Wolfe [35]), D: luer-to-tubing connector (Sigma-Aldrich, 1/16–3/32 in., cat. number: Z118028), and E: needle (BD, 22 G × 1 in., cat. number: 305155) with thin tubing (Zeus™ PTFE light wall tubing, P/N: TFT22-NT, AWG 22) connected. After sparging, the needle is removed prior to inoculation leaving the tubing in the culture. Other components include G: Nalgene™ tubing (80 PVC tubing-FDA/USPVI, 1/8 ID × 3/16 OD × 1/32 wall, Thermo Scientific cat. number: 8000-0010); H: Monoject™ needle (16 G × 1 1/2″, cat. number: 140394); and I: needle (BD, 15 G × 3 1/2 in., cat. number: 511108).

**Figure 6:**
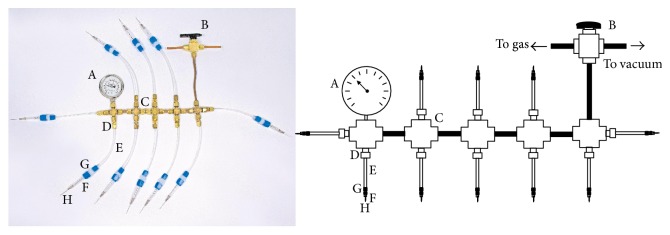
Gassing manifold for exchanging headspace of culture tubes. (A) Vacuum-pressure gauge (Swagelok, cat. number: PGI-63C-OC60-LAQX); (B) three-way valve with Swagelok fittings (Swagelok, cat. number: B-43XS4) for connection to sources of O_2_-free gas and vacuum; (C) Swagelok tube fitting, union cross, 1/4 in. (Swagelok, cat. number: B-400-4); (D) brass Swagelok tube fitting, reducer, 1/8 in. × 1/4 in. (Swagelok, cat. number: B-200-R-4); (E) thin bore polyethylene tubing, 1/8 in. (Freelin-Wade Co., cat. number: 1A-109-01); (F) PFA Swagelok tube fitting, reducing union, 1/4 × 1/8 in. (Swagelok, cat. number: PFA-420-6-2); (G) 1 cc plastic syringe barrel (BD, cat. number: 309659; cut off grip on the end of the barrel to allow insertion into the union); and (H) disposable needle (BD, 22 G × 1 in., cat. number: 305155).

**Figure 7:**
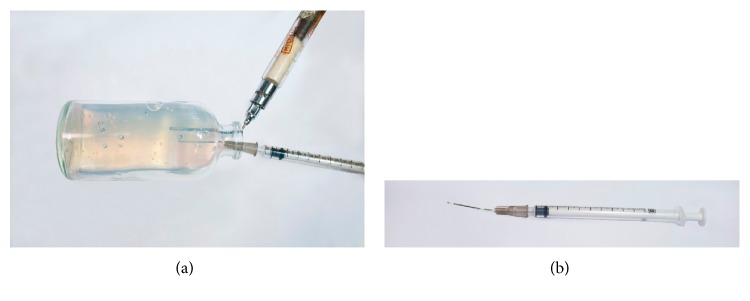
Scheme of picking colonies from agar bottles at the gassing station. (a) A 70 mL serum bottle plated with *M. maripaludis* S2 forms colonies after 3 days. After opening, a sterile N_2_ gassing cannula was introduced (a) to prevent air from entering the bottle and to maintain anaerobiosis. A 1 mL syringe with a 22 G × 1-inch needle was used for transferring the colony (b). To pick the colony more accurately, the needle was bent about 45 degrees by folding the end of the needle against the needle cap. (b) Enlarged image of the 1 mL syringe with the bent needle.

**Table 1:** Most probable number dilution of *M. maripaludis* S2 in medium prepared by the rapid protocol^a^.

	Inoculum (number of cells)	Positive number	Negative number
Three O_2_ removal cycles	1000	5	0
	100	5	0
	10	1	4
	1	1	4
	0.1	0	5

Control	1000	5	0
	100	5	0
	10	3	2
	1	1	4
	0.1	0	5

^a^Three cycles of gas exchange used in preparation of the McF medium as described in Appendix B. The inoculum was serially diluted into 1000, 100, 10, 1, and 0.1 cells. Growth was monitored for 6 days. When the OD_600 nm_ was greater than 0.6, growth was defined as positive. Control medium was prepared in the anaerobic chamber as described in Appendix A.

**Table 2:** 

Component	For tubes	For 1-liter bottle
	For 100 mL	For 1000 mL
Glass-distilled water	30 mL	300 mL
Glycylglycine buffer, 1 M, pH = 8.0	20 mL	200 mL
General salt solution	50 mL	500 mL
K_2_HPO_4_, 14 g/L	1.0 mL	10 mL
Na acetate·3H_2_O, 136 g/L	1.0 mL	10 mL
Trace mineral solution [27]	1.0 mL	10 mL
Iron stock solution [35]	0.5 mL	5 mL
Resazurin, 0.1 g/100 mL	0.1 mL	1 mL
Sodium formate (NaCOOH)	2.7 g	27 g
Sodium bicarbonate (NaHCO_3_)	0.5 g	5.0 g
Casamino acids (for complex medium)	0.5 g	5.0 g
Alanine (optional, 100 mM)	1.0 mL	10 mL

^a^Medium components are based upon Balch and Wolfe [35], Romesser et al. [36], and Whitman et al. [27].

**Table 3:** 

Component	For tubes	For 1-liter bottle
	For 100 mL	For 1000 mL
Glass-distilled water	10 mL	100 mL
Glycylglycine buffer, 1 M, pH = 6.5	40 mL	400 mL
General salt solution	50 mL	500 mL
K_2_HPO_4_, 14 g/L	1.0 mL	10 mL
Na acetate·3H_2_O, 136 g/L	1.0 mL	10 mL
Trace mineral solution	1.0 mL	10 mL
Iron stock solution	0.5 mL	5 mL
Resazurin, 0.1 g/100 mL	0.1 mL	1 mL
Sodium formate (NaCOOH)	2.7 g	27 g
Sodium bicarbonate (NaHCO_3_)	0.5 g	5.0 g
Casamino acids (for complex medium)	0.5 g	5.0 g

**Table 4:** 

Composition	g/L	Medium concentration (mM)
KCl	0.67	4.5
MgCl_2_·6H_2_O	5.50	13.5
MgSO_4_·7H_2_O	6.90	14.0
NH_4_Cl	1.00	9.0
CaCl_2_·2H_2_O	0.28	0.95

**Table 5:** 

Composition	g/L	Medium concentration (*μ*M)
Nitriloacetic acid	1.5	78
MnSO_4_·2H_2_O	0.1	5.3
Fe(NH_4_)_2_(SO_4_)_2_·H_2_O	0.2	5.1
CoCl_2_·6H_2_O	0.1	4.2
ZnSO_4_·7H_2_O	0.1	3.5
CuSO_4_·5H_2_O	0.01	0.4
NiCl_2_·6H_2_O	0.025	1.1
Na_2_SeO_3_	0.2	11.6
Na_2_MoO_4_·2H_2_O	0.1	4.1
Na_2_WO_4_·2H_2_O	0.1	3.0

## Appendix

### A. Basal Medium for Formate Growth (McF) of *Methanococcus maripaludis* and *Methanothermococcus okinawensis*

1. Select proper glassware.

This medium contains 0.4 M formate. During growth, methanogens convert the formate to CO_2_ and CH_4_ gas and cause an increase in pressure. Thus, for every liter of medium, the cells can produce about 10 liters of gas. As a result, the culture vessels can explode if the head space is not large enough to prevent an accumulation of gas pressure. For this reason, we recommend that the volume of the headspace be no less than five times the volume of medium in culture vessels that can be pressurized up to 276 kPa. Recommended maximum volumes of media for 28 mL aluminum-sealed tubes, 160 mL serum bottles, and 1 L bottles are 5 mL, 20 mL, and 100 mL, respectively. When using 1 L bottles, reduce the starting pressure of N_2_/CO_2_ to 34 kPa (see below).

(2) Deoxygenate all glassware and equipment prior to use in the anaerobic chamber. Bring culture tubes or bottles, stoppers, funnels, and pipettes and all plastic ware into the chamber at least one day before making medium to allow O_2_ to desorb or diffuse out.
(3) For medium composition^a^ see Table 2.

Combine medium ingredients and sparge with a stream of N_2_ gas for 60 minutes.

(4) Add 0.05 g cysteine-HCl or DTT per 100 mL and continue sparging for 10 minutes.
(5) Transfer medium to an anaerobic chamber, dispense medium into culture vessels and seal tubes with grey butyl stoppers (Wheaton Science Products, cat. number: W224100-202), and crimp with aluminum seals (Fisher Scientific, cat. number: 11-126-12).
(6) After removing from the anaerobic chamber, pressurize tubes and serum bottles to 103 kPa with N_2_/CO_2_ (4 : 1, *v*/*v*) and autoclave. The pH after autoclaving should be about 7.7–7.8. For one-liter bottles, pressurize to 34 kPa N_2_/CO_2_ before autoclaving. Autoclave on gravity cycle (rapid exhaust) for 20 minutes. Always autoclave these bottles secured with metal cylinders or wire baskets to limit flying glass in an explosion.
(7) Prior to inoculation, add 0.1 mL of 2.5% Na_2_S·9H_2_O (*w*/*v*) per 5 mL of medium [add 2 mL of 2.5% Na_2_S·9H_2_O (*w*/*v*) per 100 mL of medium, if made in 1-liter bottle]. For routine experiments, add the sulfide immediately before inoculation. For critical experiments, add the sulfide 8–24 h before inoculation.

#### A.1. Modifications for Growth of *Methanothermococcus okinawensis*

For cultivation of *M. okinawensis*, reduce the pH of the glycylglycine buffer to 6.5 and increase the final concentration from 0.2 M to 0.4 M. The other medium ingredients remain the same.

For medium composition see Table 3.

#### A.2. Preparation of Stock Solutions

##### A.2.1. Preparation of Glycylglycine Solution (1 M, pH = 8.0)

Use 20 mL per 100 mL of medium and the steps are as follows:

- Dissolve 132 g glycylglycine (AMRESCO, cat. number: 556-50-3) with 800 mL ddH_2_O.
- Adjust to pH 8.0 with about 18 mL of NaOH (5 M).
- Adjust volume to 1 liter. Store at 4°C in a tightly sealed bottle.

##### A.2.2. Preparation of General Salt Solution

Use 50 mL per 100 mL of medium (see Table 4).

##### A.2.3. Preparation of Iron Stock Solution [35]

Use 0.5 mL per 100 mL of medium.

To a small screw top bottle, add 0.2 g of Fe(NH_4_)_2_(SO_4_)_2_·6H_2_O and then add 2 drops of concentrated HCl followed by 100 mL of glass-distilled water.

##### A.2.4. Preparation of Trace Mineral Solution [27]

Use 10 mL per 1 liter of medium (see Table 5).

Dissolve the nitriloacetic acid in about 800 mL of diH_2_O and adjust the pH to 6.5 with KOH. Add minerals in order, allowing each one to dissolve before adding the next mineral, and adjust pH to 7.0.

##### A.2.5. Preparation of 2.5% Sodium Sulfide Solution

The steps in the preparation of 2.5% sodium sulfide solution are as follows:

- Add 100 mL ddH_2_O to a flask and mark the water line. To limit the formation of volatile hydrogen sulfide from sodium sulfide, add one pellet of NaOH (about 0.1 g). Add 10 mL more ddH_2_O to the flask.
- Boil the 110 mL ddH_2_O while flushing with N_2_ until the water level reaches the marked 100 mL water line.
- Let flask cool while flushing with N_2_ and transfer the flask to the gassing station in the fume hood. Continue to flush with N_2_.
- While flask is cooling, weigh out slightly more than 2.5 g Na_2_S·9H_2_O. Wear gloves and do the subsequent steps in the fume hood. Clean the sodium sulfide crystal by briefly rinsing the crystal with diH_2_O. To do this, swirl the crystal in a small beaker with some water until it is clean. Blot dry the crystal with paper towel. Reweigh the crystal to ensure that the final weight is 90–110% of the desired weight.
- Add the cleaned and weighted sodium sulfide to the cooled flask while flushing with N_2_ and mix until partially dissolved.
- Stopper the flask, discontinue flushing, and transfer to the anaerobic chamber. Dispense into 5 mL aliquots in 28 mL aluminum-sealed tubes.
- Remove tubes from chamber and pressure with N_2_ at 103 kPa.
- Autoclave on gravity cycle (rapid exhaust) for 20 minutes.
- Store these sodium sulfide tubes in anaerobic chamber. They should remain good for one to two months. Discard if a precipitant forms.

##### A.2.6. Preparation of 25% Sodium Sulfide Solution

The steps for the preparation of 25% sodium sulfide solution are as follows:

- Prepare as above but with 25 g Na_2_S·9H_2_O. Dispense into 5 mL aliquots in 28 mL aluminum-sealed tubes. For cultures of 0.5, 1.0, and 1.5 L, use 1.0, 2.0, and 3.0 mL, respectively.
- Store these sodium sulfide tubes in anaerobic chamber. They should remain good for two months. Discard if a precipitant forms.

### B. Solid Medium for Formate Growth (McF) of *Methanococcus maripaludis*

This protocol prepares 1% agar medium in 70 mL serum bottles for plating *M. maripaludis* and is modified from Tumbula et al. [18].

1. Solid medium is prepared with 1% agar (*w*/*v*), adding 10 mL of medium to a 70 mL serum bottle. Add 0.1 g agar into each individual serum bottle prior to medium preparation. The remaining ingredients are the same as for the broth.
2. Transfer the broth medium (without sulfide) and the agar-containing bottles to the anaerobic chamber, dispense medium into agar bottles, seal the bottles with grey butyl stoppers (Wheaton Science Products, cat. number: W224100-202), and crimp with aluminum seals (Fisher Scientific, cat. number: 11-126-12).
3. Remove the sealed bottles from the anaerobic chamber, pressurize each bottle to 103 kPa with N_2_/CO_2_ (4 : 1, *v*/*v*), and autoclave. Autoclave on gravity cycle (rapid exhaust) for 20 minutes. After autoclaving, allow the bottles to cool to touch and add sulfide to a final concentration of 2 mM. Mix gently and allow the agar bottles to cool completely on their sides for at least five hours (overnight solidification works best).
4. To obtain individual colonies of *M. maripaludis*, make a serial dilution in broth culture to about 100 cells/mL. Plate the diluted and undiluted culture (positive control) by adding 0.5 mL of the culture suspension to agar bottles using 1 mL syringes. Add the liquid to the agar surface, with the bottle resting on its side. Gently swirl the agar bottle to allow the culture suspension to become evenly distributed on the agar surface. Rest the agar bottle on its side for another four hours to allow the culture suspension to be fully absorbed into the agar. Make sure that the agar surface is flat and sitting horizontally; otherwise, the colonies will only form on one side of the agar.
5. Incubate the plated bottles by standing them upright at 37°C. Hence, the water that forms during incubation will drain to the bottom of the bottle.
6. Colonies will appear after three to five days of incubation. Because of the liquid collecting at the bottom of the bottle, confluent growth will occur on the bottom 1 cm. Avoid shaking the bottle during incubation to prevent inadvertent inoculation of the upper portion of the agar.
7. To pick individual colonies into broth in the anaerobic chamber, add 2 mM sulfide (sulfide stock preparation see Appendix A) into tubes of broth 24 hours before use. Open the stopper of the agar bottle in the anaerobic chamber, sterilize the opening with an electric heating element, and insert a 1 mL syringe into the agar bottle, carefully touching the top of a single colony with the needle. Then, stab the needle into a tube of broth and flush the syringe with medium two times to introduce some cells into the broth. Full growth is often observed after 1-2 days.

### C. Medium-Scale Cultivation System for *M. maripaludis* and *M. okinawensis*

This protocol is suitable for growth of 1.5 L cultures on formate medium using glassware modified to allow venting of the gas produced.

#### C.1. Day 1: Medium Preparation and Degassing

1. In preparation of the inoculum, one day before initiating the medium-scale culture, inoculate 0.3 mL of fresh culture into one or more serum bottles containing 30 mL of formate medium. Incubate without shaking at the proper temperature.
2. Dispense 1.5 L McF medium into a modified 2 L storage bottle (Figure 5).
3. To sparge the medium after autoclaving, insert a 2 cc interchangeable glass-filter syringe (Micro-Mate, cat. number: 14-825-1B) with luer-to-tubing connector (Sigma-Aldrich, 1/16–3/32 in., cat. number: Z118028) into a butyl rubber stopper (Bellco Glass Inc., Vineland, NJ, cat. number: 2048-11800). To the tip of the inserted needle, attach approximately 20 cm of thin tubing (Zeus PTFE light wall tubing, P/N: TFT22-NT, AWG 22, LOT number: 623928). Insert the stopper into the side arm of the culture vessel. The stopper is not sealed in place so that it can fall out and release any pressure that might form in case the exhaust lines become clogged.
4. With the cap of the 2 L bottle loosely attached, autoclave this assembly together with the gas trap (165 mL serum bottle, sealed with blue butyl rubber stopper and crimped with aluminum seals), tubing, and syringes that connect the medium bottle, gas trap, and water trap.
5. After autoclaving, allow the sterile medium to cool to at least 90°C before assembling the gassing and vent lines. First, begin sparging N_2_/CO_2_ (4 : 1 *v*/*v*) through the medium at a flow rate of ~25 mL/min. At the same time, connect the medium bottle to the gas trap and water trap. Add about 300 mL of water to the water trap. Close the cap of the 2 L bottle to begin gas flow through the exhaust system.
6. Sparging should exchange the headspace of the bottle at least ten times. At a flow rate of 25 mL/min, the medium should be sparged for 200 min or about 5 L of gas.
7. While sparging, add cysteine hydrochloride to a final concentration of 0.5 g L^−1^. To prepare this cysteine hydrochloride stock solution, affix a sterile 0.2 *μ*M filter (Whatman™, cat. number: 6780-2502) to a 5 mL syringe with Luer-Lok tip (BD™, cat. number: 309646) with the plunger removed. Add 3 ml ddH_2_O to the syringe and then add 0.75 g of cysteine hydrochloride (Sigma, cat. number: 52-89-1). Swirl the solution gently to fully dissolve the cysteine. Put a needle (BD, 15 G × 3 1/2 in., cat. number: 511108) onto the tip of the 0.2 *μ*M filter and quickly inject the freshly made cysteine hydrochloride solution into the 1.5 L culture bottle through the side-arm stopper.
8. When the gassing is complete and the medium has cooled to the growth temperature or below, incubate the entire assembly at the growth temperature, 37°C for *M. maripaludis* or 62°C for *M. okinawensis*, overnight.

#### C.2. Day 2: Inoculation and Following Incubation

1. The next morning, add sodium sulfide to 2 mM with a syringe through the side arm. It is often convenient to add 3 mL of a 25% (*w*/*v*) sterile solution prepared as in Appendix A. Continue to incubate the medium at the growth temperature for another six hours to allow the medium to become fully reduced.
2. Inoculate the bottle with 30 mL of culture (approximately OD_600 nm_ = 0.6) from day 1. Overnight or about 15 h, *M. maripaludis* S2 can grow to an OD_600 nm_ = 0.8 at 37°C. Similarly, *M. okinawensis* IH1 can grow to about OD_600 nm_ = 0.6 at 62°C.

### D. Rapid Preparation of Formate Medium for Growing *Methanococcus maripaludis*

This rapid protocol is designed for laboratories that have limited anaerobic equipment. All procedures can be performed without using an anaerobic chamber.

#### D.1. Liquid Medium Preparation

1. On the bench, combine all medium ingredients as described in Appendix A. Add the cysteine hydrochloride (0.05 g/100 mL) without sparging the medium shortly before dispensing the medium into tubes.
2. Dispense medium into aluminum-sealed tubes aerobically. Seal each tube with a grey butyl stopper (Wheaton Science Products, cat. number: W224100-202) and crimp with aluminum seals (Fisher Scientific, cat. number: 11-126-12).
3. Bring the sealed tubes to a gassing station as shown in Figure 6. Run three gas-exchange cycles on each tube. Each cycle is composed of 45 s of vacuum followed by 15 s of pressurization at 103 kPa by N_2_/CO_2_ (4 : 1, *v*/*v*)_._ At this point, the medium may still be pink because the resazurin is not reduced. Upon autoclaving, the medium should become colorless. If using serum bottles with larger volumes, increase the cycle times. For instance, 160 mL serum bottles, 90 s vacuum and 30 s pressurization are sufficient for a system with a strong vacuum line.
4. Autoclave on gravity cycle (rapid exhaust) for 20 min.

#### D.2. Solid Medium Preparation

The rapid protocol prepares 1% agar medium in 70 mL serum bottles.

On the bench, combine all medium ingredients as described in Appendix A and add the cysteine hydrochloride (0.05 g/100 mL) without sparging the medium but shortly before dispensing into the bottles.

1. Solid medium is prepared with 1% agar (*w*/*v*) to 10 mL of medium in a 70 mL serum bottle. Add 0.1 g agar into each serum bottle prior to medium preparation.
2. Dispense medium into agar-containing serum bottles aerobically. Seal each bottle with a grey butyl stopper (Wheaton Science Products, cat. number: W224100-202) and crimp with aluminum seals (Fisher Scientific, cat. number: 11-126-12).
3. Bring sealed bottle to the gassing station as shown in Figure 6. Run three gas-exchange cycles on each bottle, each cycle is composed of 45 s of vacuum followed by 15 s of pressurization to 103 kPa by N_2_/CO_2_ (4 : 1, *v*/*v*)_._ At this point, the medium may still be pink because the resazurin is not reduced. Upon autoclaving, the medium will become colorless.
4. Autoclave on gravity cycle (rapid exhaust) for 20 min. After autoclaving, allow the bottles to cool to touch, using the gassing station to anaerobically add sodium sulfide at a final concentration of 2 mM (for sulfide stock solution preparation, see Appendix A). Mix gently and then allow the agar bottles to cool completely on their sides for at least five hours (overnight solidification works best).

#### D.3. Plating and Picking Colonies on the Bench

The Hungate technique [33] is used to make anaerobic transfers at the gassing station. Briefly, before a transfer, flame-sterilize a cannula and begin the flow of N_2_ gas. Flush a sterile syringe with N_2_ by inserting the needle into the cannula and pumping the syringe three times to remove air. Fill the syringe with N_2_ gas. After sterilizing the top of the stopper, immediately expel the N_2_ from the syringe while pushing the needle through the stopper. This procedure removes any air that was introduced into the tip of the needle.

1. Plating is performed as described in Appendix B steps 4–6, but using the gassing station for all anaerobic transfers.
2. To pick individual colonies into broth, use the Hungate technique [33]. At the gassing station, remove the aluminum crimp-sealed stopper from the agar bottle and flame the opening. To keep the serum bottle O_2_-free, introduce a flow of N_2_ gas with a sterile N_2_ gassing cannula (Figure 7(a)). To transfer a colony, use a 1 mL syringe and 22 Ga × 1-inch needle. Bend the needle about 45 degrees by folding the end of the needle against the needle cap. Be sure that the hole on the tip of the needle is on the bottom side after the bend (Figure 7(b)). This makes it easier to collect a colony inside the needle tip. Insert this syringe into the agar bottle, pumping the plunger a few times to fill the syringe with N_2_. With the syringe barrel pulled back about 1 cm, carefully touch the top of a single colony with the needle (Figure 7(a)). Withdraw the syringe from the bottle and stab the needle into a tube of broth medium. Flush the syringe two times with 0.2 mL of medium to wash the cells from the needle. Full growth is often observed after 1-2 days.
